# High gain quad-port circularly polarized aperture-coupled MIMO antenna

**DOI:** 10.1038/s41598-025-19993-6

**Published:** 2025-09-18

**Authors:** Abdelhady M. Abdelhady, Hesham A. Mohamed, Wael A.E. Ali, Ahmed A. Ibrahim

**Affiliations:** 1 Benha Faculty of Engineering, Benha University, Benha, Egypt; 2 Electronics Research Institute, Microstrip Circuits Joseph Tito St, Huckstep, El Nozha, Cairo, 11843 Egypt; 3 Department of Electronics & Communications Engineering, College of Engineering and Technology, Arab Academy for Science, Technology and Maritime Transport (AASTMT), Alexandria, Egypt; 4 Electronics and Communications Engineering Dep, Minia University, El-Minia, Egypt; 5 Communications and Computer Engineering Department, Nahda University, Benisuef, Egypt

**Keywords:** Circular polarization, Aperture-coupled, MIMO antenna, Axial ratio, Engineering, Physics

## Abstract

This paper presents the design and implementation of a quad-port aperture-coupled circularly polarized (CP) multiple-input multiple-output (MIMO) antenna, specifically fitted for X-band applications. The proposed antenna features an innovative design that uses a 90° power divider to excite two orthogonal modes. This is achieved through an aperture Line that is coupled to a patch via two unique, orthogonal dog-bone slots. A key element of the design is a parasitic patch, which incorporates a 9× 9 array of square pixel cells. This array is strategically included to simultaneously boost the antenna’s gain and improve its axial ratio (AR) bandwidth. The problem of achieving high-performance CP MIMO in the X-band is addressed by the clever use of orthogonal mode excitation and the parasitic patch with its array. By carefully coupling the orthogonal modes, the antenna achieves circular polarization, which is critical for reducing multipath interference. The parasitic patch’s role is to act as a director, enhancing the overall radiation characteristics without significantly increasing the antenna’s size or complexity. This method allows for a compact design while maintaining high performance. The experimental and simulation results showed strong agreement. The antenna achieved an impressive impedance bandwidth (IBW) of 9.8–13 GHz (28%) and a 3-dB axial ratio bandwidth (ARBW) of 10.7–12.27 GHz (13.67%). Measured port isolations are below − 20 dB, with a gain of 7–8 dBic across the frequency band. The antenna’s MIMO performance was excellent, as evidenced by a diversity gain (DG) of at least 9.99 dB, an envelope correlation coefficient (ECC) of 0.002 or less, and channel capacity loss (CCL) ≤ 0.2 bit/s/Hz, demonstrating its outstanding diversity capabilities. These results confirm the antenna’s suitability for advanced X-band wireless technologies that require high data rates and robust connectivity.

## Introduction

 In modernistic wireless technologies, multiple input multiple output (MIMO) system is significantly used to enhance the channel capacity and the use of circularly polarized (CP) antennas for multipath propagations and line-of-sight communications^1^. CP antennas generate radio waves with a constantly rotating electric field, mitigating signal fading caused by misalignment between antennas^2,3^. By meticulously designing the radiating patches, feed network, and coupling slots, engineers can achieve CP in aperture-coupled MIMO antennas. This combination offers reduced signal fading since CP ensures robust communication even with antenna misalignment, crucial in MIMO systems^4^. Also, aperture-coupled feeding allows for a low-profile antenna suitable for various applications with reduced inherent coupling between feed and radiating elements in aperture-coupling to minimize interference between MIMO antenna ports^5^.

An aperture-coupled MIMO antenna with circular polarization is a type of MIMO antenna that uses an aperture-coupled feed structure to achieve circular polarization with broadband matching. The antenna consists of two or more radiating elements that are coupled through a slot in the ground plane, which allows for the excitation of circularly polarized waves^6–8^. The use of circular polarization can provide improved immunity to multipath fading and reduce the impact of shadowing, making it suitable for applications such as wireless local area networks (WLANs) and satellite communications^9^. The aperture-coupled design also allows for compact and lightweight implementation, making it ideal for handheld devices and other space-constrained applications^10^. Moreover, aperture-coupled feeding uses a slot or aperture in the ground structure to electromagnetically couple the feed line with the radiator. This offers advantages such as reduced fabrication complexity, wider bandwidth compared to traditional feeding, and improved isolation between the feed and radiating elements^11^. Aperture-coupled microstrip patch antennas with circular polarization (CP) offer a compelling solution for MIMO systems. It’s worth mentioning that DRA aperture-coupled feeding antennas feature compactness, broadband matching, and wide-band CP performance^12–14^.

The CP MIMO antennas can be utilized in different applications, such as WLAN, where MIMO with CP antennas enhances signal reception for mobile devices in various orientations^14^. Furthermore, it can be used in 5G and beyond (6G) communication systems, where the high data rates and spatial diversity requirements of future communication systems can be addressed by MIMO with CP antennas^15^. Additionally, MIMO with CP antennas can improve data transmission reliability for satellite communication links^16^. There are some challenges and considerations in designing MIMO antennas with CP and aperture-coupled feeding, since they can be intricate compared to simpler antenna designs. Also, achieving a wide bandwidth for both impedance and axial ratio (AR) is considered one of the main challenges in the suggested design, taking into consideration the optimization of the antenna design for specific MIMO configurations^17–19^.

Recently, various works have been published focusing on circularly polarized MIMO antennas, and among these reported works are the following: in^20^, the authors presented a two-port wide-band and highly isolated CP MIMO antenna, and the performance is improved by using defected ground structure (DGS) and parasitic elements, which are mounted in different layers with the radiator. It achieved a wide bandwidth of 5.1–5.6 GHz with a gain value better than 8 dBi and an isolation ranging from 30 to 63 dB in the achieved frequency band. The authors in^21^ introduced a dual-circularly polarized antenna, and a hybrid coupler is utilized to generate dual radiation polarization. The 4 × 4 array is designed at 5.5 GHz and then fabricated with isolation better than 14 dB, axial ratio 29.1% (4.70–6.30 GHz), and impedance bandwidth 4.66–6.31/(30%) below − 10 dB. A four-port dual-band (0.902–0.928 and 2.4–2.485 GHz) circularly polarized MIMO antenna is introduced in^22^. The MIMO antenna is comprised of four inverted-F monopole antennas with a 90^o^ phase-delay feeding network, and the achieved isolation is above 20 dB for the 1 st frequency band and over 25 dB for the 2nd frequency band. Furthermore, the obtained gain and axial ratio values for the 1 st band are 4.1 dBi and 14.2% and for the 2nd band, 7.4 dBi and 7.4%, respectively. In^23^, a 2-port circularly polarized MIMO antenna is suggested for C-band application using parasitic elements and truncated patches to generate dual–sense circular polarization. Moreover, the two-port MIMO antenna succeeded in obtaining the same impedance bandwidth (IBW) and AR bandwidth (ARBW) of 5.0–5.6 (11.3%), isolation between ports higher than 32 dB in the obtained frequency band, and the gain results vary from 6 to 8.5 dBi. In order to circularly polarized behavior with 2 2-port MIMO antenna for sub-6 GHz 5G NR, a modified L-shaped rectangular patch with optimized DGS loaded with Z-shaped slot using machine learning algorithms to obtain the optimized ARBW in^24^. The impedance bandwidth for both ports is 3–4.2 GHz with an IBW% % of 31.5%, and the achieved ARBW is 2.6–3.9 GHz, and it’s a percentage of 40% with isolation between ports exceeding 25 dB. The authors in^25^ proposed a frequency-selective structure (FSS) based 2-port CP MIMO antenna. The antenna is operated at 7.6–11.6 GHz with isolation between ports of more than 25 dB with an AR below 3 dB between 9.2 and 9.8 GHz. The antenna gain is improved after incorporating the FSS by 21% over the achieved frequency band, as well as the radiation efficiency, with values > 84.2%. In^26^, a 2-port MIMO antenna with a T-divider for dual circular polarization WLAN applications is presented. The antenna succeeded in achieving dual polarization in the range 2.43–2.485 GHz with coupling less than − 10 dB and maximum achievable gain of 8 dBi. The authors in^27^ introduced a 2-port CP MIMO dielectric resonator antenna (DRA). The suggested antenna is operated in the range 5.5–8.0 GHz with a BW% of 37.1% and ARBW of 6.0 to 6.55 GHz with isolation exceeding 21.8 dB. In^28^, the authors presented a two-port wide-band CP MIMO antenna, and the performance is improved by using an octal-shaped monopole antenna with etched partial ground planes for CP obtainment purposes. It achieved a wide bandwidth of 2–11.2 GHz with a gain of around 7.87 dBi, and isolation exceeds 21.7 dB in the achieved frequency band.

This paper introduces a circularly polarized antenna with an aperture-coupled feed for X-band applications. The suggested antenna consists of 4 layers, and the antenna succeeded in obtaining the required band from 10 to 13.5 GHz with an AR of 17.4%. The antenna is extended to support MIMO communications by presenting a 4-port model with orthogonal arrangement between each two neighbor elements to improve the isolation between ports (≥ 20 dB). The radiation characteristics are also implemented to ensure the diverse capability of the suggested MIMO antenna; also, the proposed CP antenna is fabricated and measured to validate the simulation outcomes. By leveraging the advantages of aperture-coupled feeding and circular polarization, MIMO antennas can achieve improved performance and robustness in various wireless communication applications.

## The simulation outcomes

### The unit antenna design

**Fig. 1:**
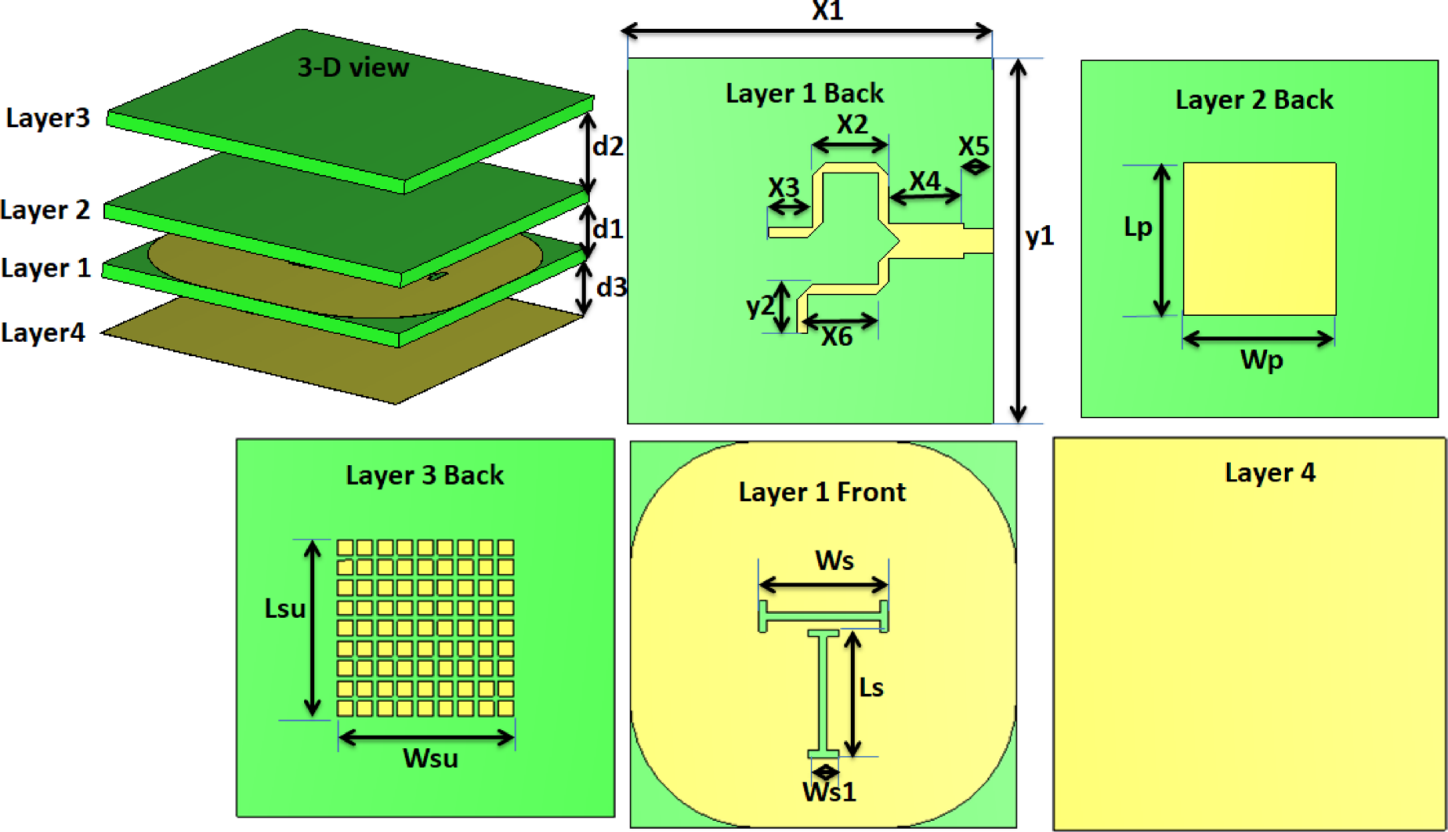
The single antenna configuration with d_1_ = 2.03 mm, d_2_ = 3.55 mm, d_3_ = 2.5 mm, X_1_ = 15 mm, X_2_ = 3.2 mm, X_3_ = 1.8 mm, X_4_ = 3.1 mm, X_5_ = 1.2 mm, X_6_ = 2.85 mm, Y_1_ = 15 mm, Y_2_ = 1.4 mm, W_s_= 5 mm, L_s_ =5 mm, W_s1_= 1.2 mm, L_p_= 6.4 mm, W_p_= 6.4 mm, Lsu = 7 mm, Wsu = 7 mm.

The proposed single antenna is shown in Fig. 1. It is designed to generate circular polarization (CP) by exciting two orthogonal modes. These orthogonal modes are achieved by feeding the patch with a 90^°^ power divider, as shown in Fig. 1 (layer 1 back). The 90° power divider was implemented using two striplines with a quarter-wavelength length difference. Theoretically, both striplines have a 3 dB power level with a 90° phase difference. The power divider was first simulated with port #1 as the input and ports #2 and #3 as outputs. The phase difference remains close to 90° around 11.9 GHz. The power divider’s arms are ended by two bent stubs for improving the matching and minimizing mutual coupling. The main patch radiator in layer 2 is fed by an aperture-coupled technique through two orthogonal dog-bone slots, as shown in Fig. 1 (layer 1 front). These two slots are etched on a modified-shaped ground for better isolation. 9 × 9 small rectangular cells are utilized as a parasitic element to improve the gain and AR bandwidth simultaneously, as shown in layer 3. Finally, the investigated antenna is backed with a solid copper sheet as shown in Fig. 1 (layer 4) to operate as a reflector for enhancing the antenna gain and reducing the radiated field pattern back lobe.

**Fig. 2:**
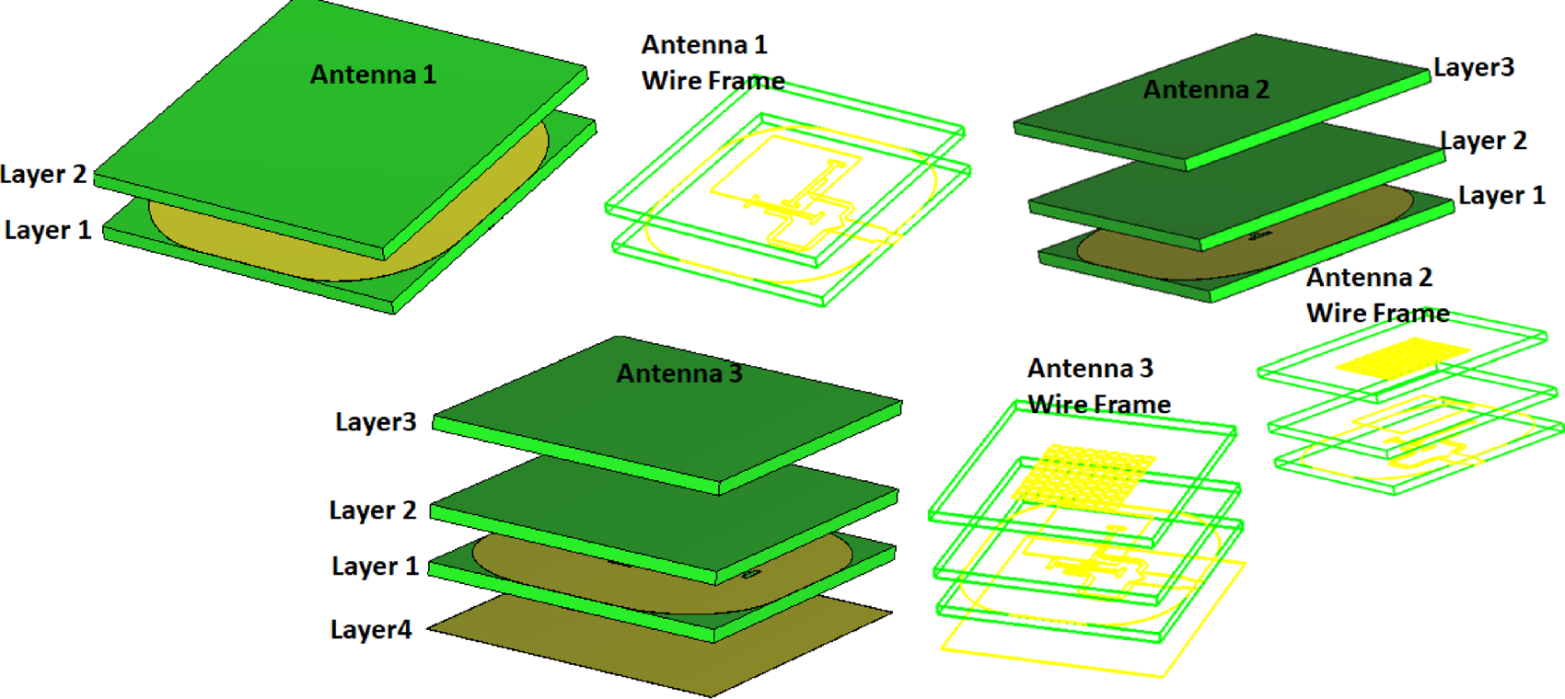
The single antenna design steps.

**Fig. 3:**
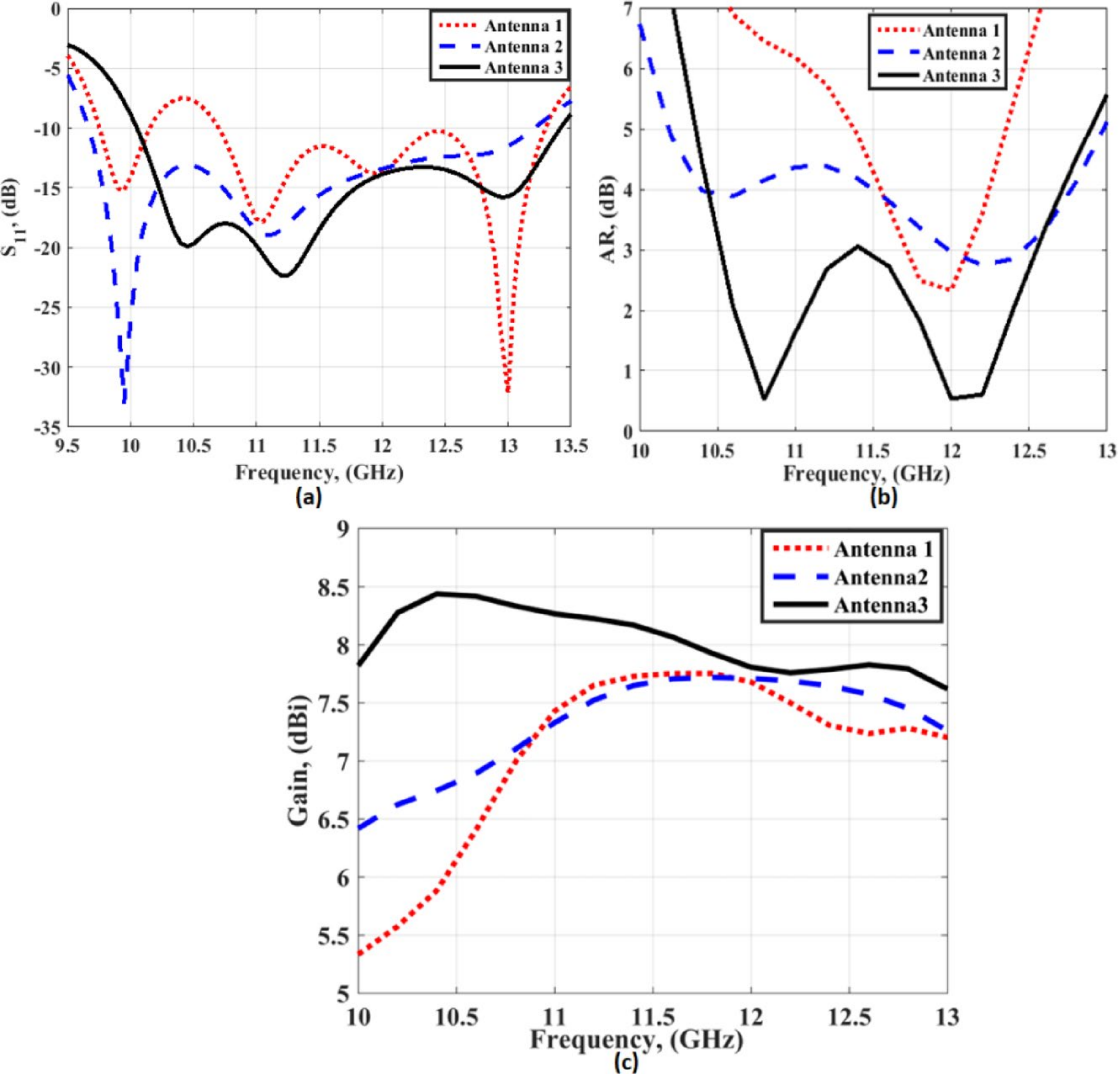
The single antenna design steps outcomes (**a**) S_11,_ (**b**) AR, (**c**) Gain.

The single antenna design steps are utilized to investigate the effect of each layer on the return loss and the AR, and the gain, as demonstrated in Fig. 2. The first antenna (antenna 1) is composed of two layers. The antenna is an aperture coupled feed and the power is coupled to the patch in layer 2 through two orthogonal dog-bone slots as depicted in Fig. 2. The antenna is operated from 9.7 GHz to 10.1 GHz and from 10.6 to 13.4 GHz with S_11_ ≤ −10 dB and the 3-dB AR from 11.7 GHz to 12.1 GHz (3.36%) and the gain is varied from 5.5 dBi at 10 GHz to 7.5 dBi at 11.5 GHz as displayed in Fig. 3 (the red dotted line). Antenna 2 is composed of three layers, as shown in Fig. 2. 9 × 9 small rectangular cells are added to the aperture antenna as presented in Fig. 2, antenna 2. The antenna is utilized from 9.6 GHz to 13.4 GHz with S_11_ ≤ −10 dB, and the 3-dB AR from 12 GHz to 12.5 GHz (4.08%), and the gain is varied from 6.5 dBi at 10 GHz to 7.5 dBi from11GHz to 13 GHz as displayed in Fig. 3 in the blue dashed Line. Finally, the suggested antenna is designed by applying a complete copper sheet as layer 4, as shown in Fig. 2, to operate as a reflector for enhancing both the antenna gain and extending the AR bandwidth. It is operated from 10 GHz to 13.54 GHz with S_11_ ≤ −10 dB and the 3-dB AR from 10.5 GHz to 12.5 GHz (17.39%), and the gain is varied from 8.5 dBi at 10 GHz to around 8 dBi from 11 GHz to 13 GHz, as displayed in Fig. 3, the black solid line. In conclusion, the parasitic patch array and metallic ground improve gain, axial ratio, impedance matching, and radiation pattern performance.

**Fig. 4:**
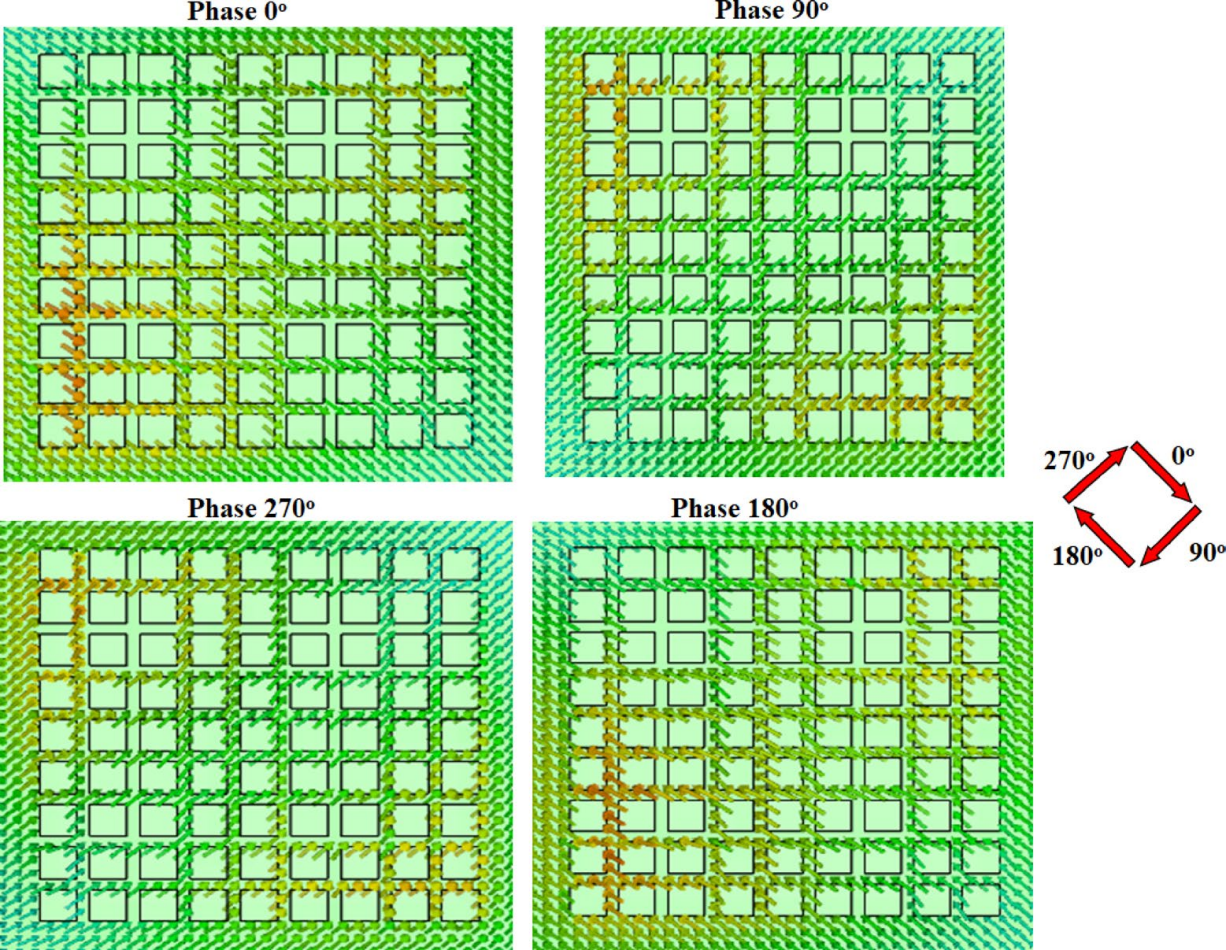
Surface current distribution at 10.6 GHz.

**Fig. 5:**
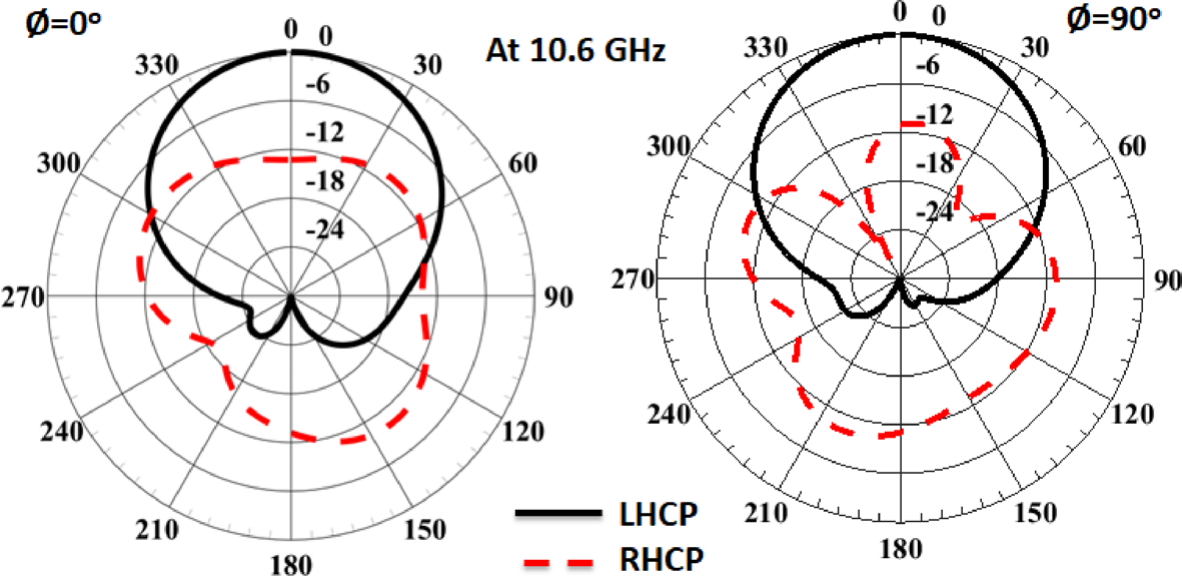
The single antenna co (LHCP) and cross-polarization (RHCP) at 10.6 GHz.

To confirm the CP response of the suggested antenna, the current distribution at different phases at 10.6 GHz is shown in Fig. 4. It is noticed that the antenna behaves like LHCP. The left-hand (LH) and the right-hand (RH) CP patterns of the single antenna at 10.6 GHz are illustrated in Fig. 5. The antenna has a directive pattern in the broadside direction with a maximum value at 0^o^. As well, the antenna achieved LHCP with more than 13 dB difference over the RHCP cross-polarization component.

### The proposed MIMO antenna

**Fig. 6:**
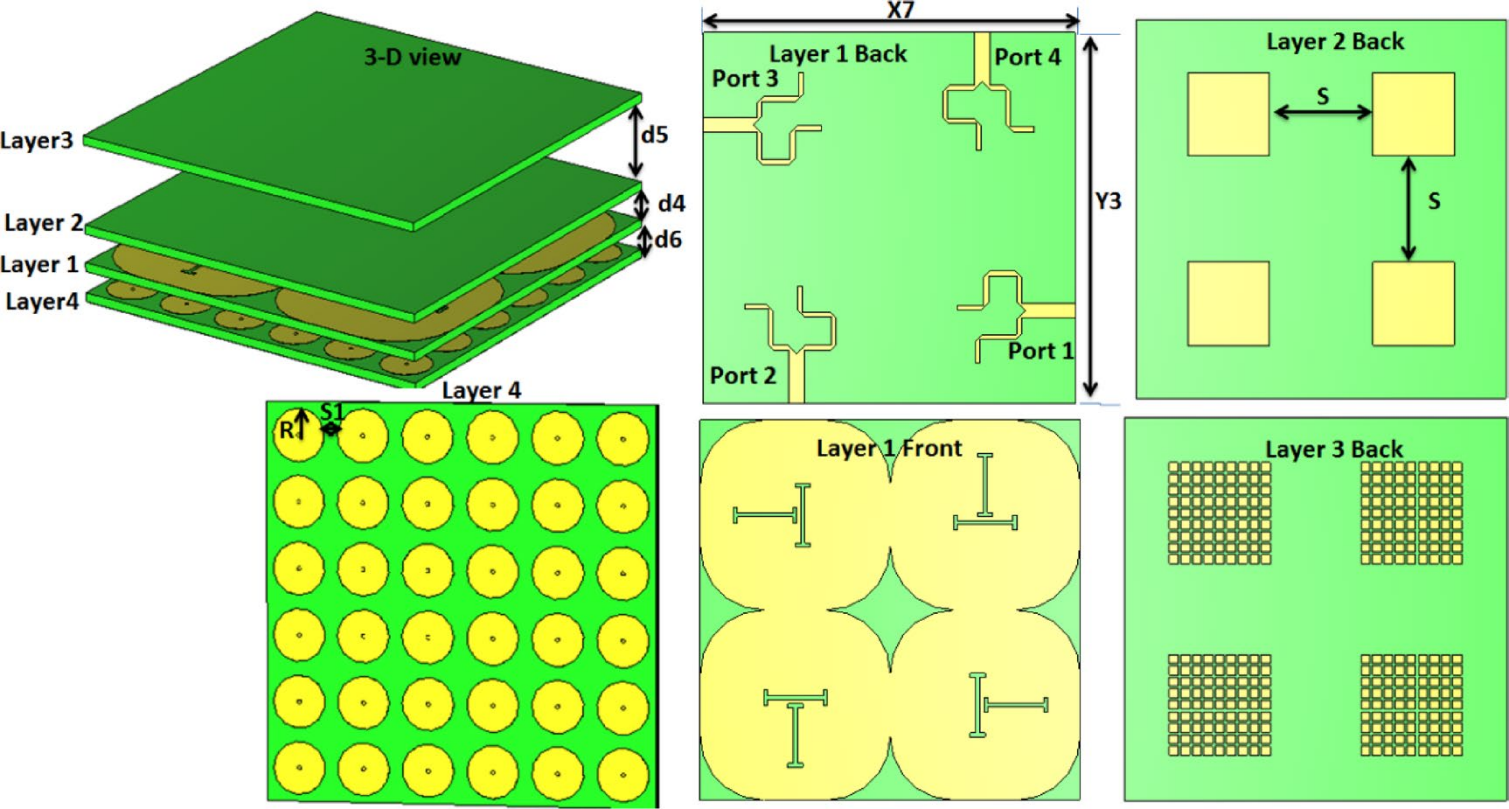
The MIMO antenna configuration with d_4_ = 2.03 mm, d_5_ = 5.7 mm, d_6_ = 1.51 mm, X_7_ = 30 mm, Y_3_ = 30 mm, *R* = 2 mm, S = 8.4 mm, s_1_ = 1 mm.

The presented CP single antenna in the previous section is repeated four times to compose the suggested MIMO antenna by keeping λ/4 separation between elements to achieve the desired outcomes without changing the radiation characteristics of the single antenna as illustrated in Fig. 6. Rogers (RO3006) with a permittivity of 6.15, tan δ = 0.002 and a thickness of 0.635 mm is utilized as the building block of the substrate of the four layers. The suggested antenna has dimensions of 30 × 30 × 11.79 mm^³^. The complete copper sheet (layer 4) of the previous section is replaced by a 6 × 6 electromagnetic band gap (EBG) as shown in Fig. 6 (layer 4). The EBG-shaped mushroom cells are composed of circular discs on the top of the substrate and connected to a copper sheet in the rear of the substrate through metallic vias for antenna gain improvement. The four antenna elements sharing the same ground and with orthogonal ports, the configuration satisfies the intended isolation between ports with no need for any isolation structure. Also, the orientation of the antenna elements is chosen to guarantee that the CP performance is similar to the single element.

**Fig. 7:**
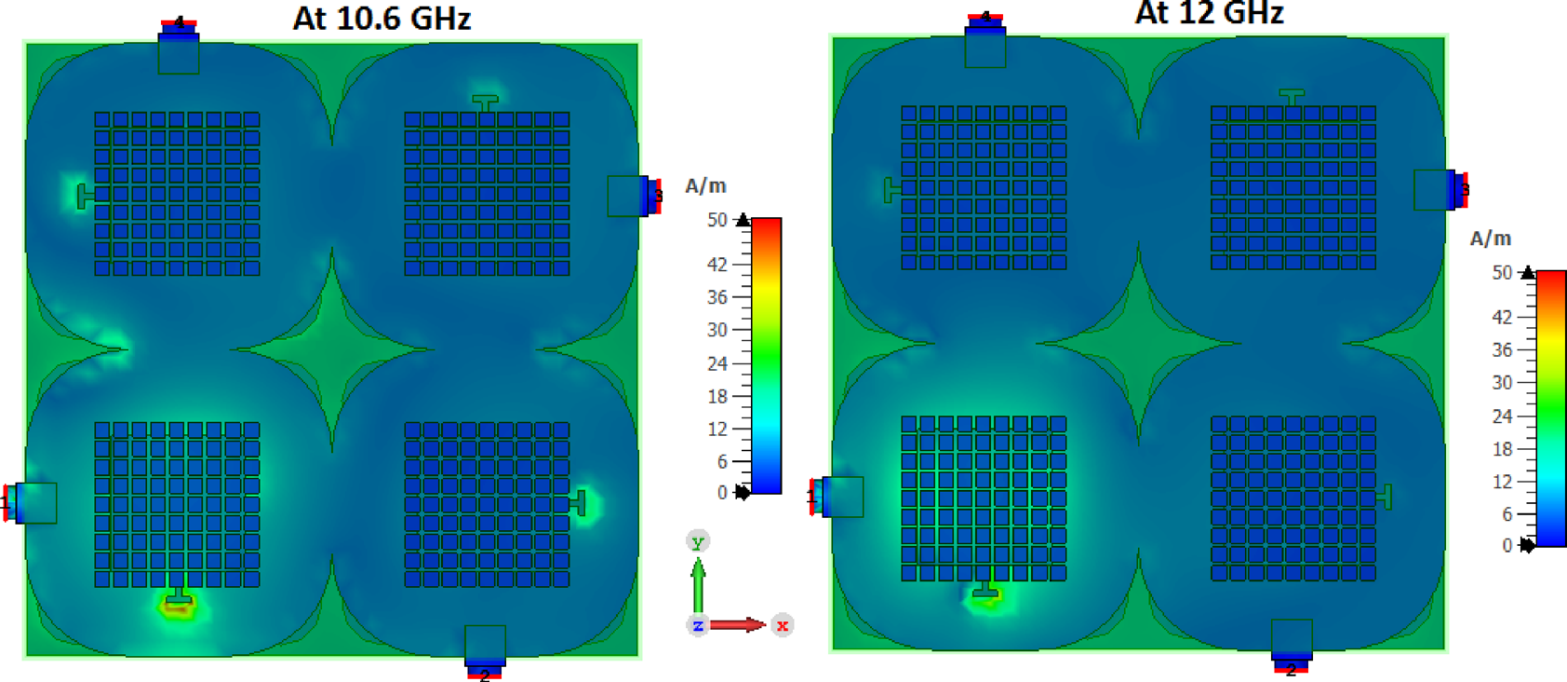
The current distributions at port 1.

Figure 7 demonstrates the current distribution of the MIMO antenna at 10.6 GHz and 12 GHz. It is observed that there is a small current transferred from port 1 to other ports, which confirms the high isolation between ports without using an extra decoupling structure.

## Experimental outcomes

### Single unit

**Fig. 8:**
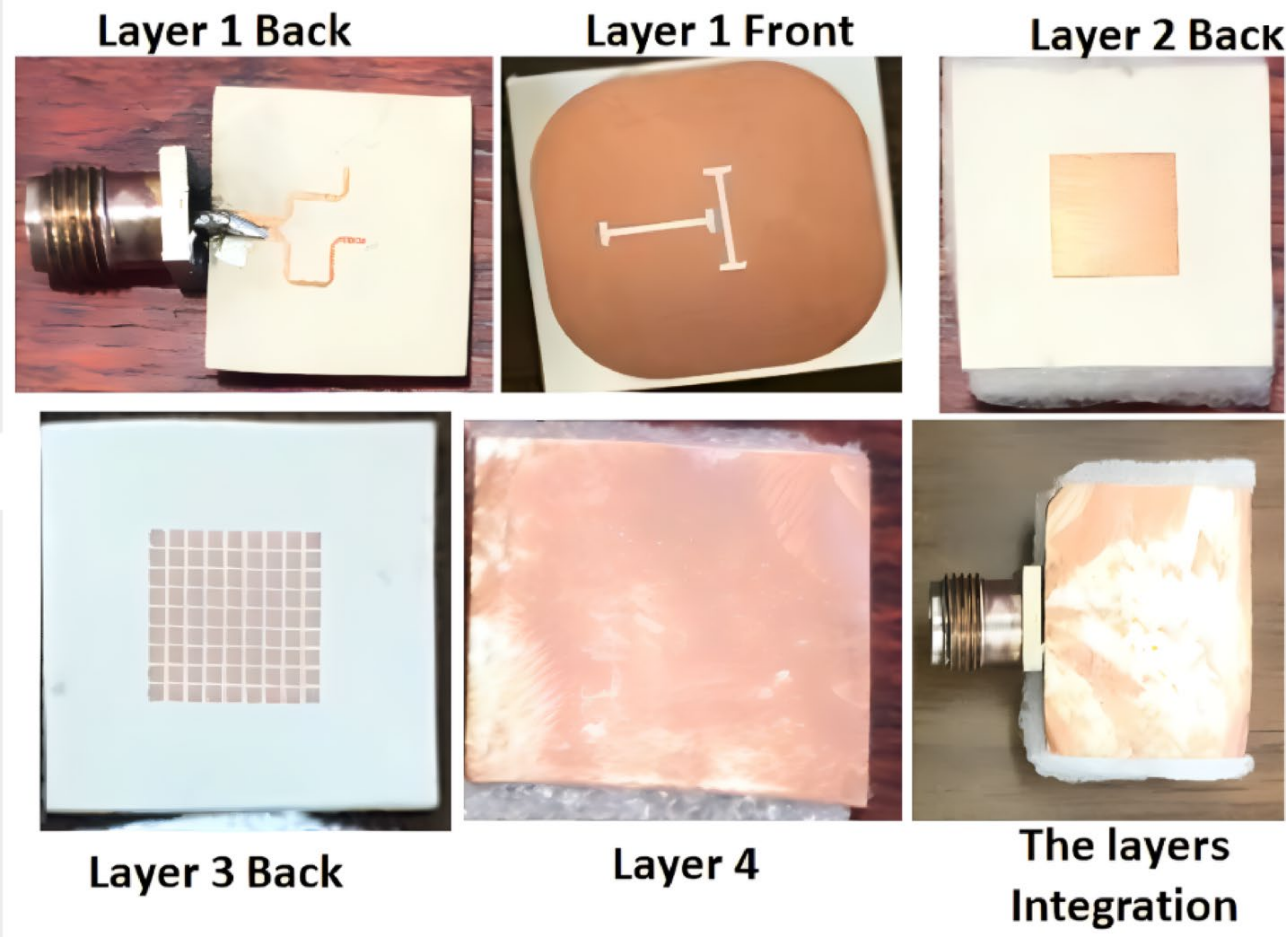
The single antenna layers fabrication photos.

The antenna is fabricated, tested, and its layer’s fabrication photos are shown in Fig. 8. Rogers (RO3006) with a permittivity of 6.15, loss tangent = 0.002, and 0.635 mm thickness is utilized as the building substrate of the three layers.

**Fig. 9:**
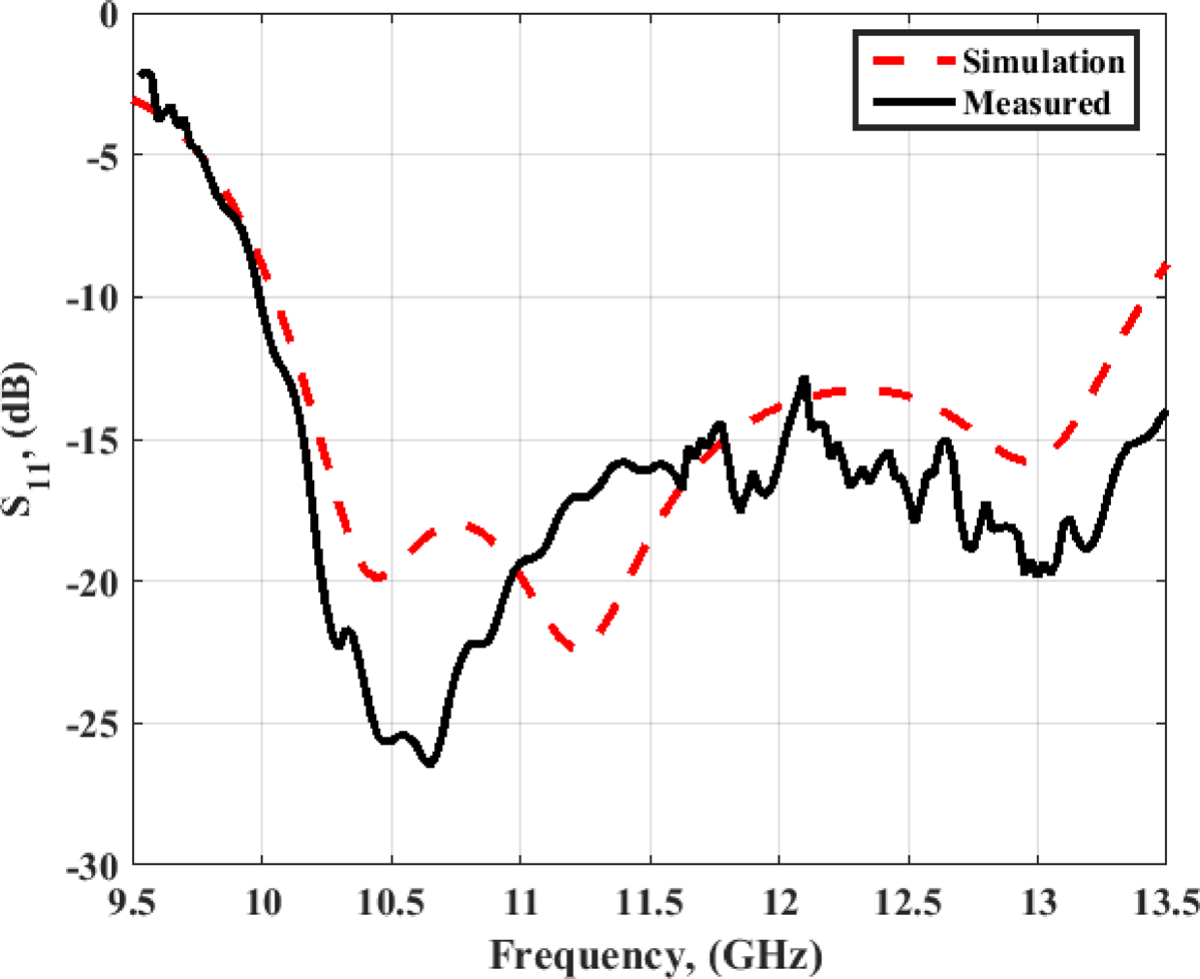
The single antenna simulated and measured outcomes of S_11_.

The vector network analyzer (VNA) ZVA 67 is used in the validation process. The antenna S_11_ is demonstrated in Fig. 9. The antenna has the same trend between the simulated and examined outcomes with S_11_ ≤ −10 dB from 10 up to 13.5 GHz (29.7%).

**Fig. 10:**
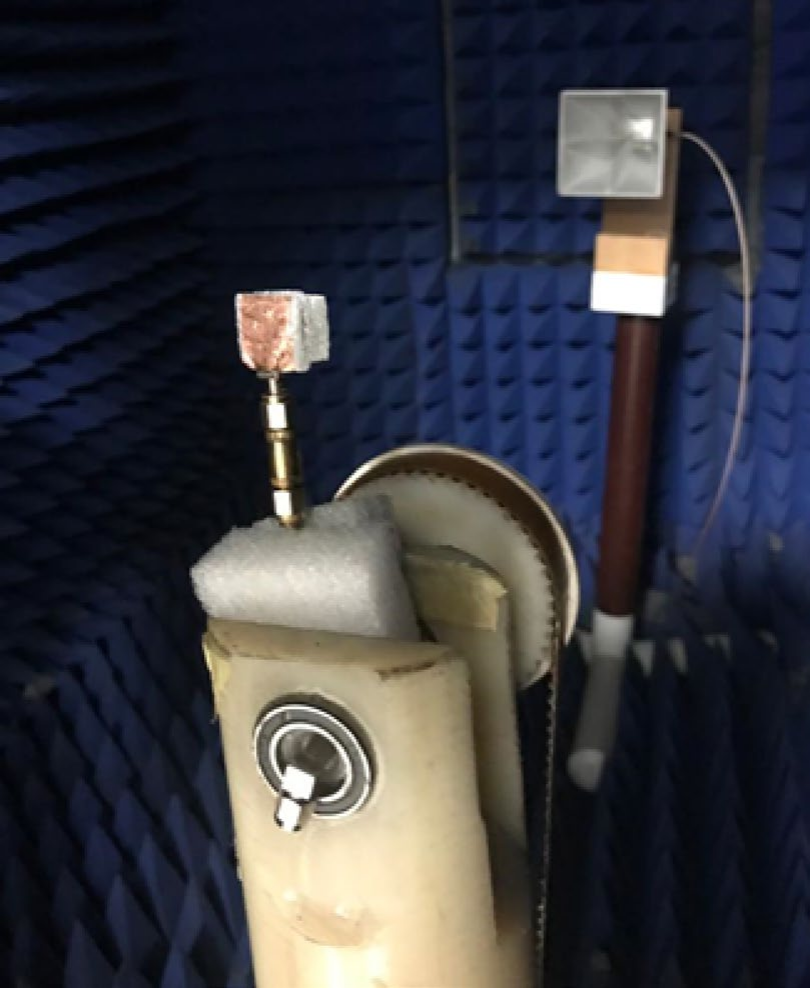
The single antenna radiation patterns testing setup.

**Fig. 11:**
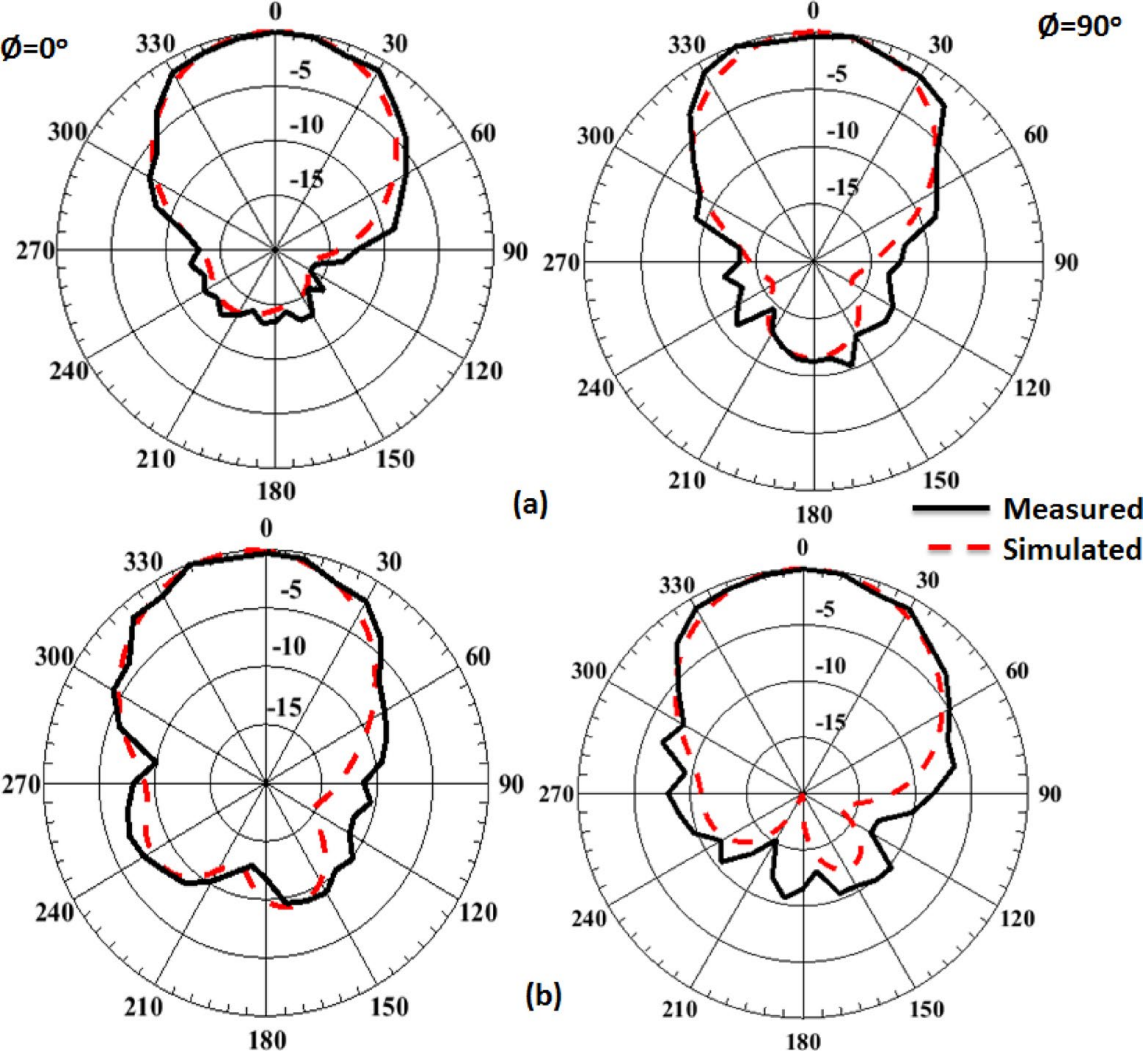
The single antenna normalized simulation and measurement patterns (a) at 10 GHz and (b) at 12 GHz.

The single antenna is validated in the anechoic chamber to obtain its radiation patterns. The setup for testing the radiation patterns is demonstrated in Fig. 10. Figure 11 illustrates the normalized simulation and practical radiation patterns of the single antenna at 10 GHz and 12 GHz. It is noticed that the antenna has a directed beam at 0^o^ (broadside direction). Moreover, there is an outstanding match between the simulated and tested outcomes.

### Four-port MIMO antenna

**Fig. 12:**
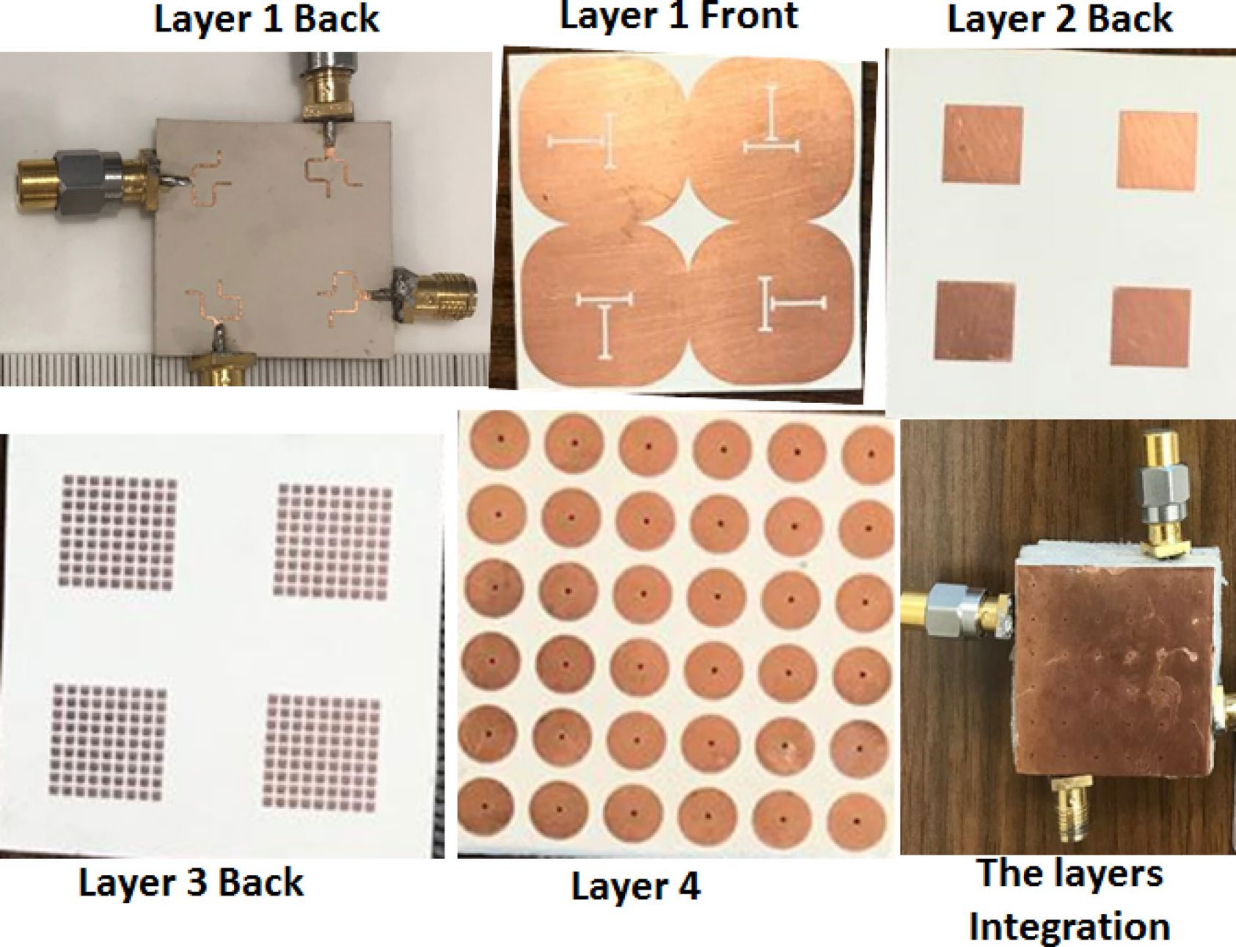
The MIMO antenna layers fabrication photos.

The suggested MIMO antenna is fabricated and tested, and its layers’ fabrication photos are depicted in Fig. 12.

**Fig. 13:**
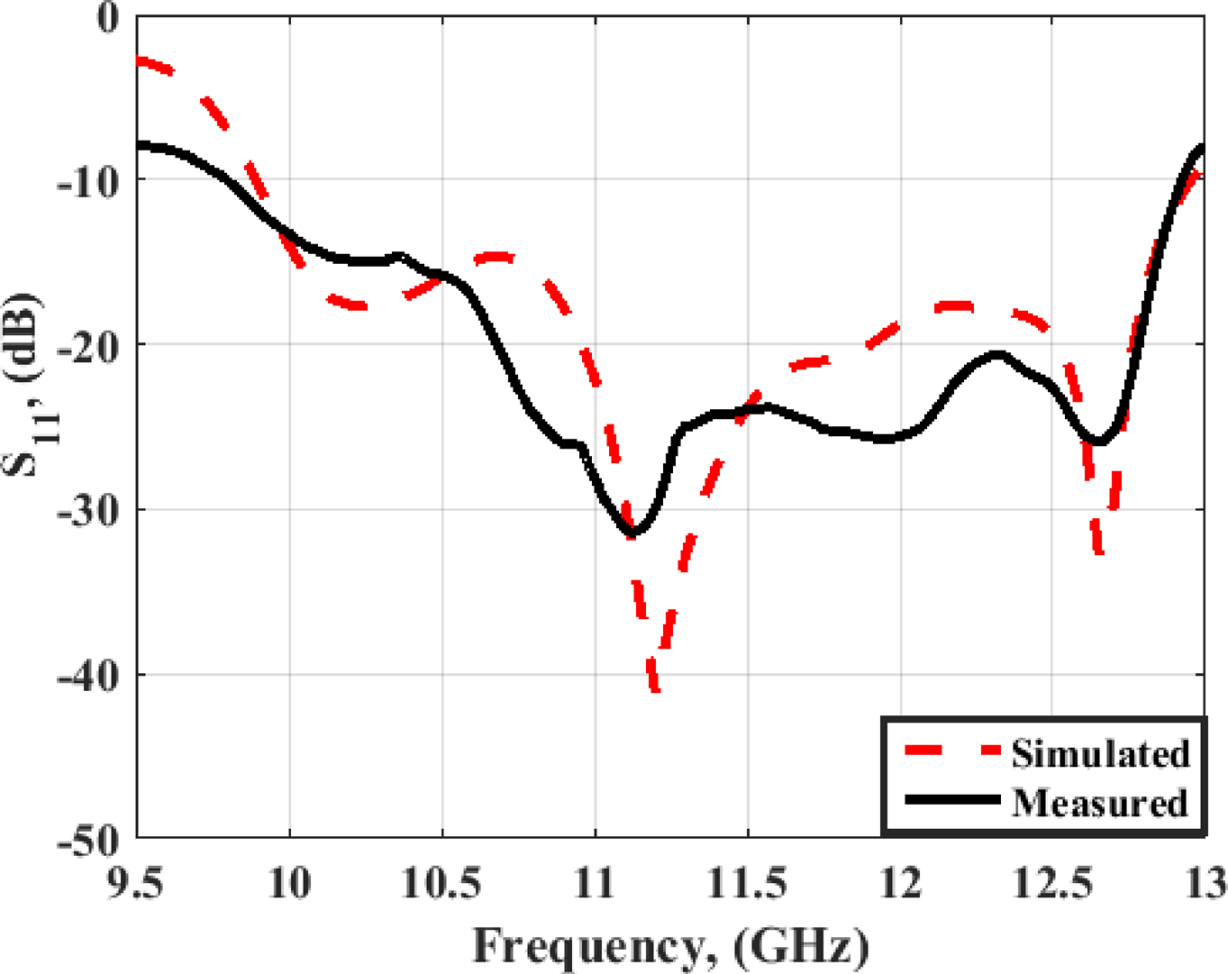
The MIMO antenna simulation and measurement outcomes of S_11_ at port 1.

The VNA ZVA 67 is used in the validation process. The antenna S_11_ is demonstrated in Fig. 13. The antenna has outstanding matching between the simulated and measured results with S_11_ ≤ −10 dB from 9.8 up to 13 GHz (28%).

**Fig. 14:**
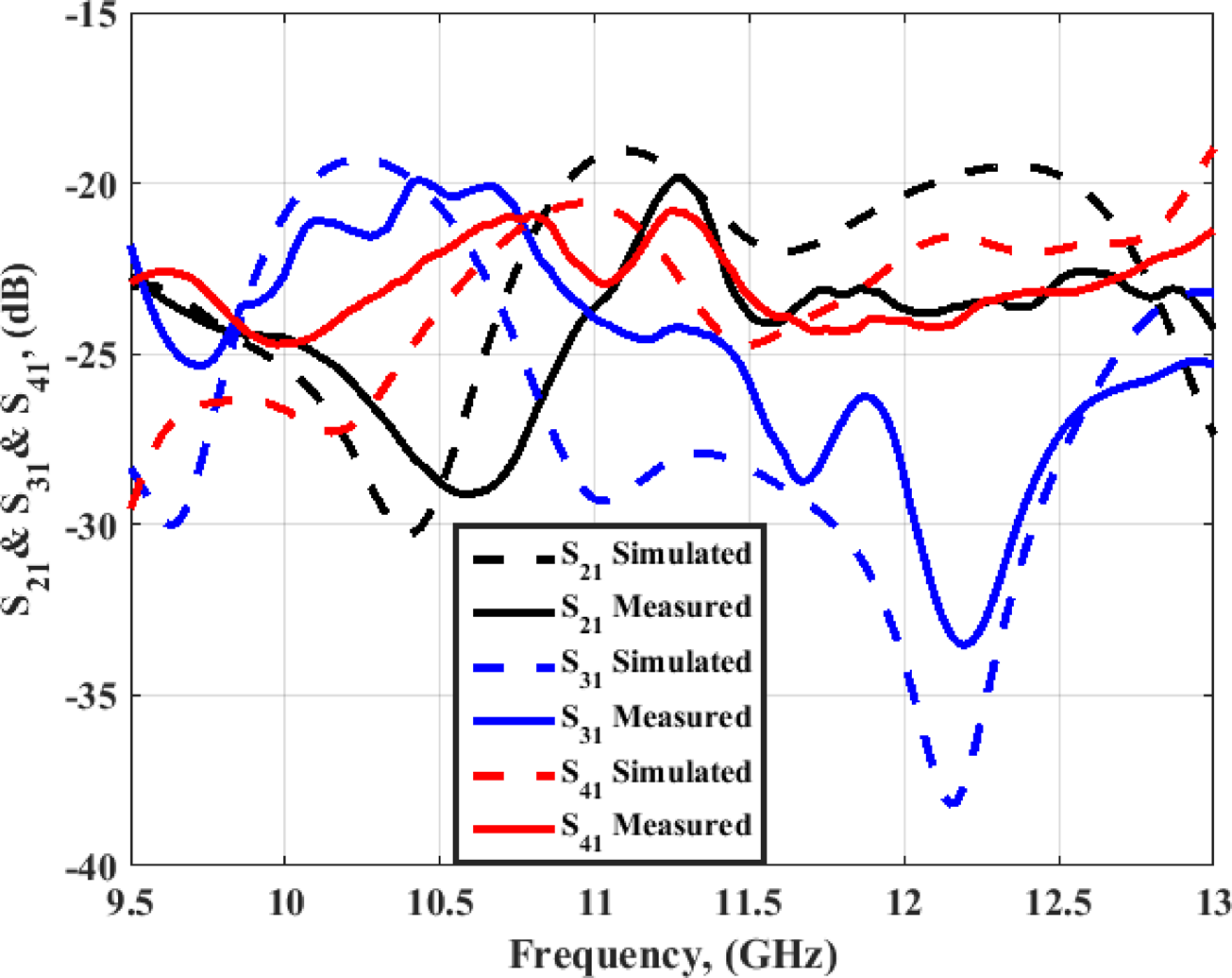
The MIMO antenna simulation and measurement outcomes of S_21_, S_31_, and S_41_.

The isolation of the antenna is calculated and tested at port 1 as a function of (S_21_, S_31_, and S_41_) as demonstrated in Fig. 14. The antenna has achieved mutual coupling less than − 20 dB within the operating frequency band, which confirms the high isolation between elements. The differences between the results are due to the fabrication and measurement process. As well, there are human errors during the alignment between the four layers. However, the outcomes have the same trend.

**Fig. 15:**
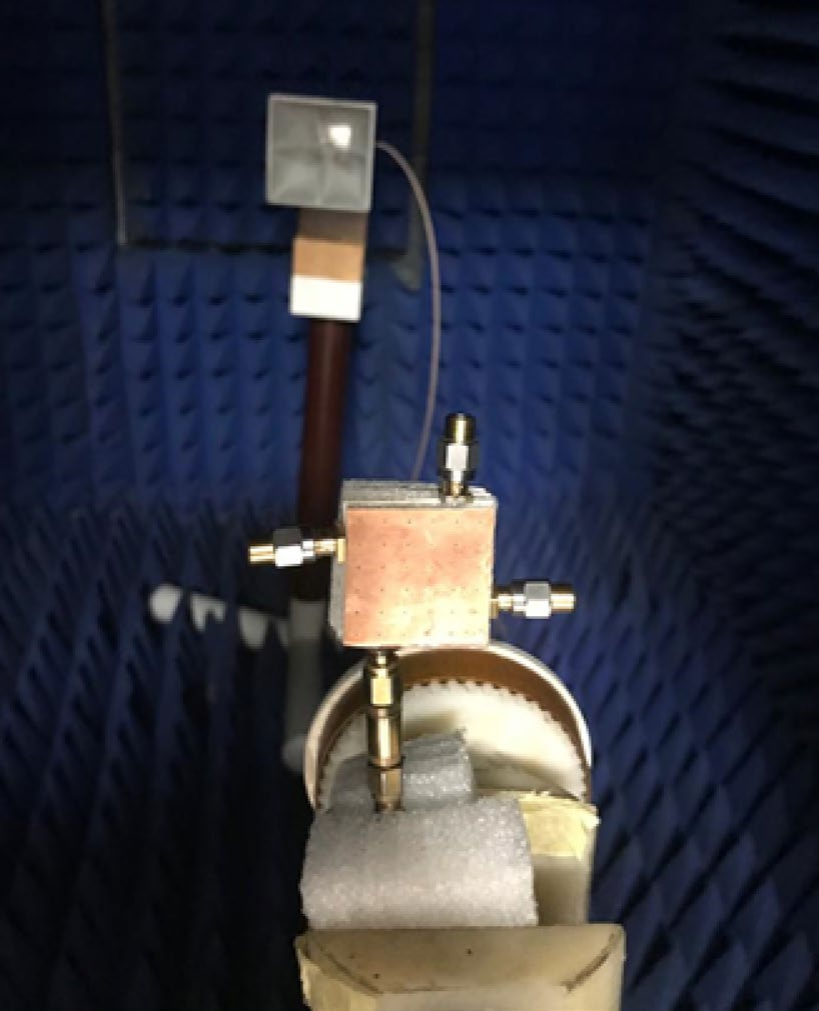
The MIMO antenna radiation patterns testing setup.

**Fig. 16:**
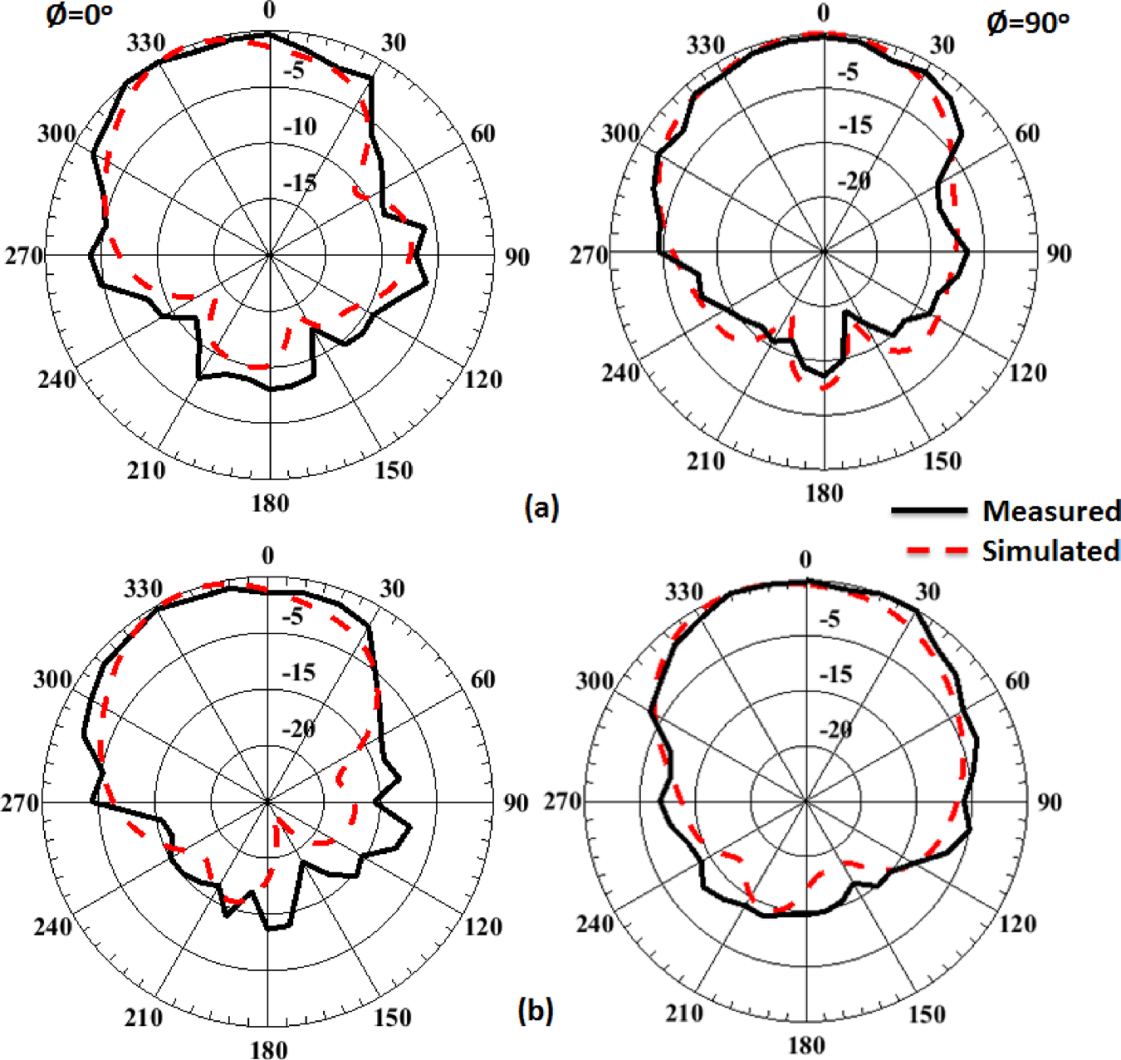
MIMO boresight normalized simulation and measurement field patterns.

The MIMO antenna is experimented with in the anechoic chamber to obtain its radiation patterns when excited at port 1. The setup for testing the radiation patterns is illustrated in Fig. 15. As the MIMO patterns are expected to be broadside directive patterns, it is aligned with their maximum facing the horn aperture for measuring their radiated fields. Figure 16 illustrates the normalized patterns of the MIMO antenna at port 1 at 10 GHz and 12 GHz. It is seen that a good match between both outcomes.

(a) at 10 GHz and (b) at 12 GHz.

**Fig. 17:**
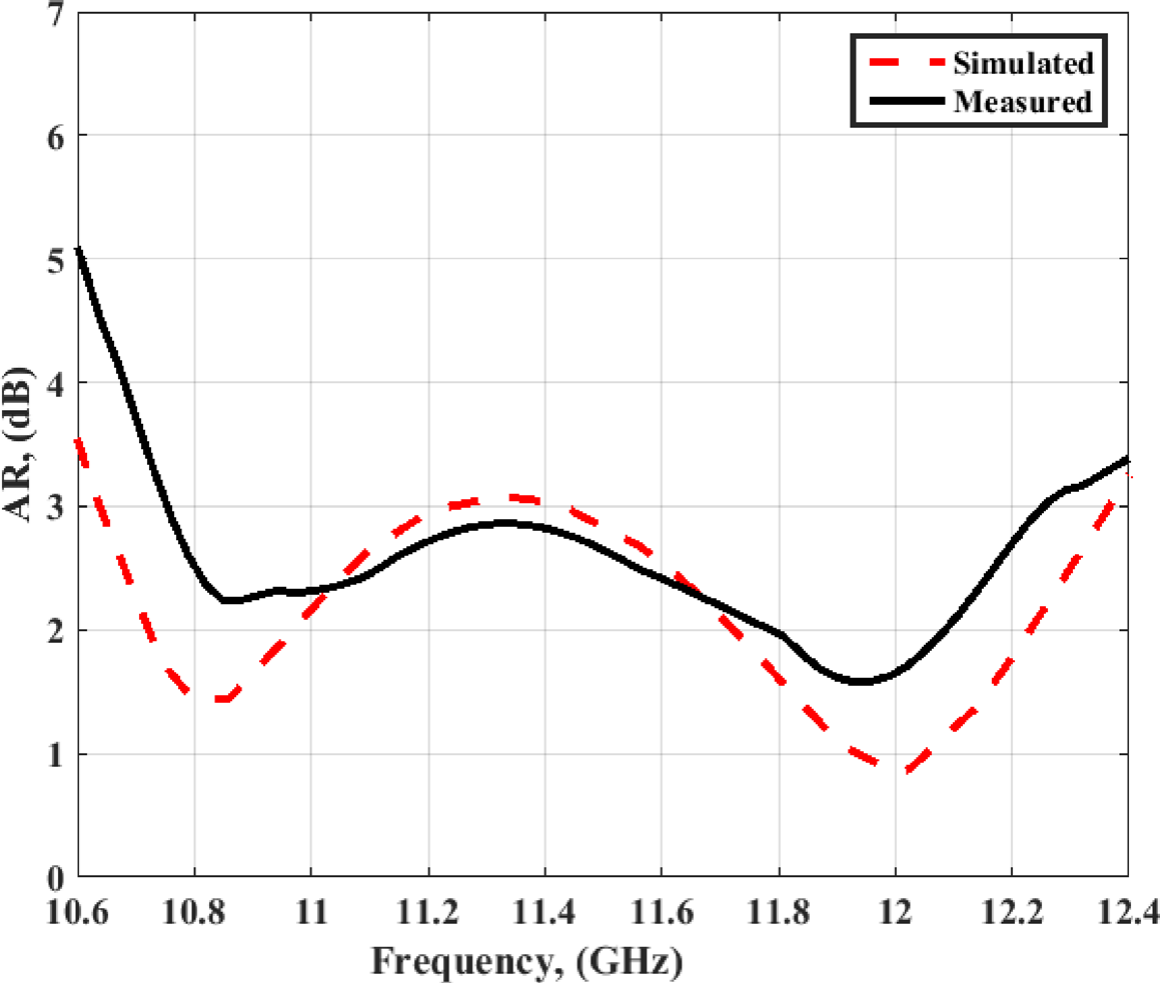
The MIMO antenna simulated and measured AR at port 1.

**Fig. 18:**
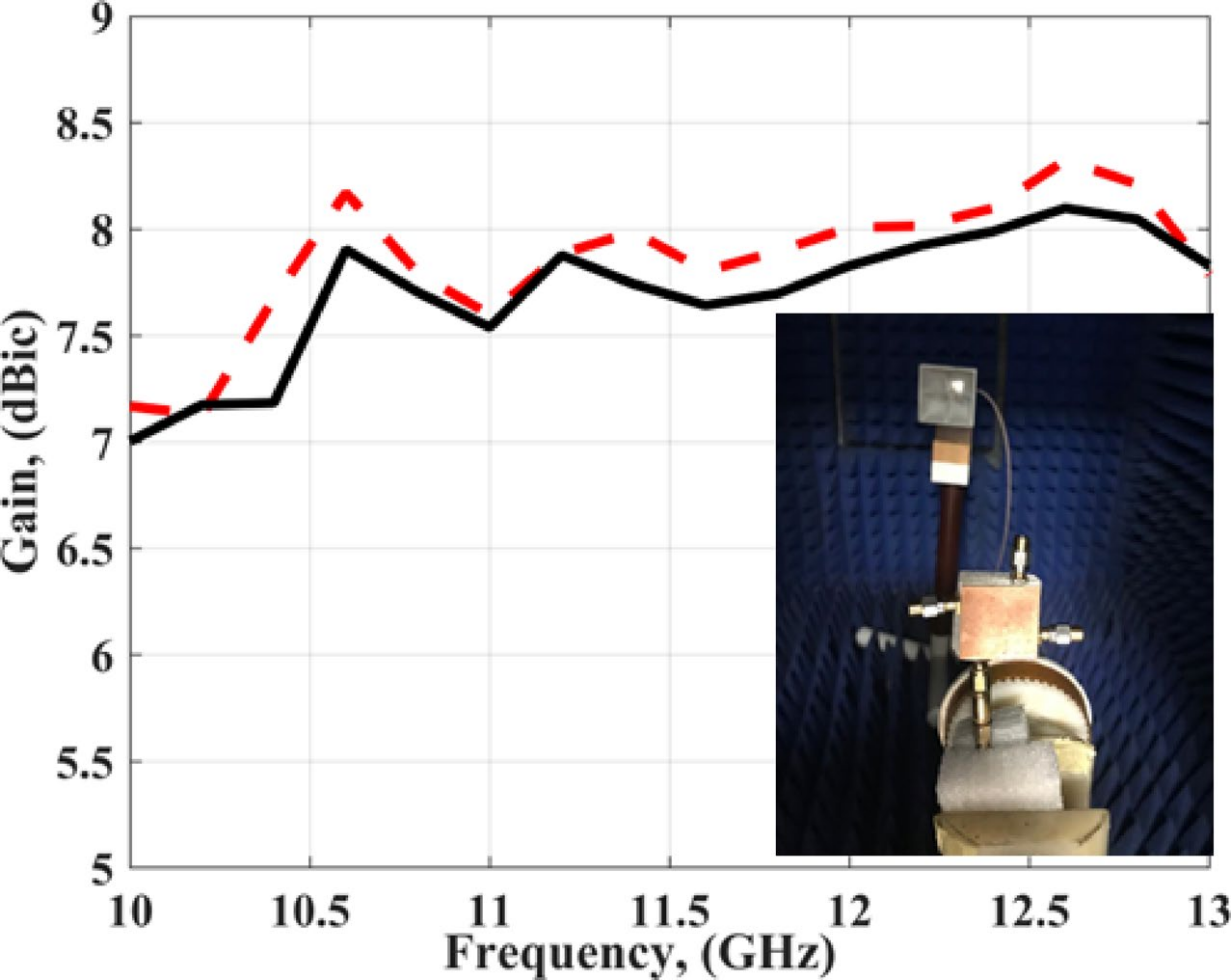
The MIMO antenna simulated and the gain at port 1.

The AR simulated and tested outcome at port 1 is displayed in Fig. 17. The antenna has simulated a 3-dB AR extended from 10.6 GHz to 12.4 GHz (15.6%), while it achieved a measured 3-dB AR extended from 10.7 GHz to 12.27 GHz (13.67%). The differences between the outcomes of the AR are due to the fabrication and measurement tolerance. As well, there are human errors during the alignment between the four layers. However, the outcomes still have good matching. The antenna simulated and tested gain at port 1 is demonstrated in Fig. 18. The antenna has a gain varied from 7 dBic to 8 dBic within the required frequency band.

To investigate the performance of the MIMO antenna, several parameters such as ECC, DG, and CCL are calculated and extracted. The ECC is used to show the correlation between antenna elements. The ECC with a lower value is preferred because it means the correlation between elements is small, which achieves high MIMO performance. The ECC can be calculated and extracted from Eq. 1 for S-parameters^29–32^, and the equations used from^29,30^ for the radiation patterns.

1 $$ECC = {\rho _e}(i,j,N) = \frac{{{{\left| {\sum\limits_{n = 1}^N {S_{i,n}^*{S_{n,j}}} } \right|}^2}}}{{{\prod _{k = i,j}}\left[ {1 - \sum\limits_{n = 1}^N {S_{k,n}^*{S_{n,k}}} } \right]}}$$

**Fig. 19:**
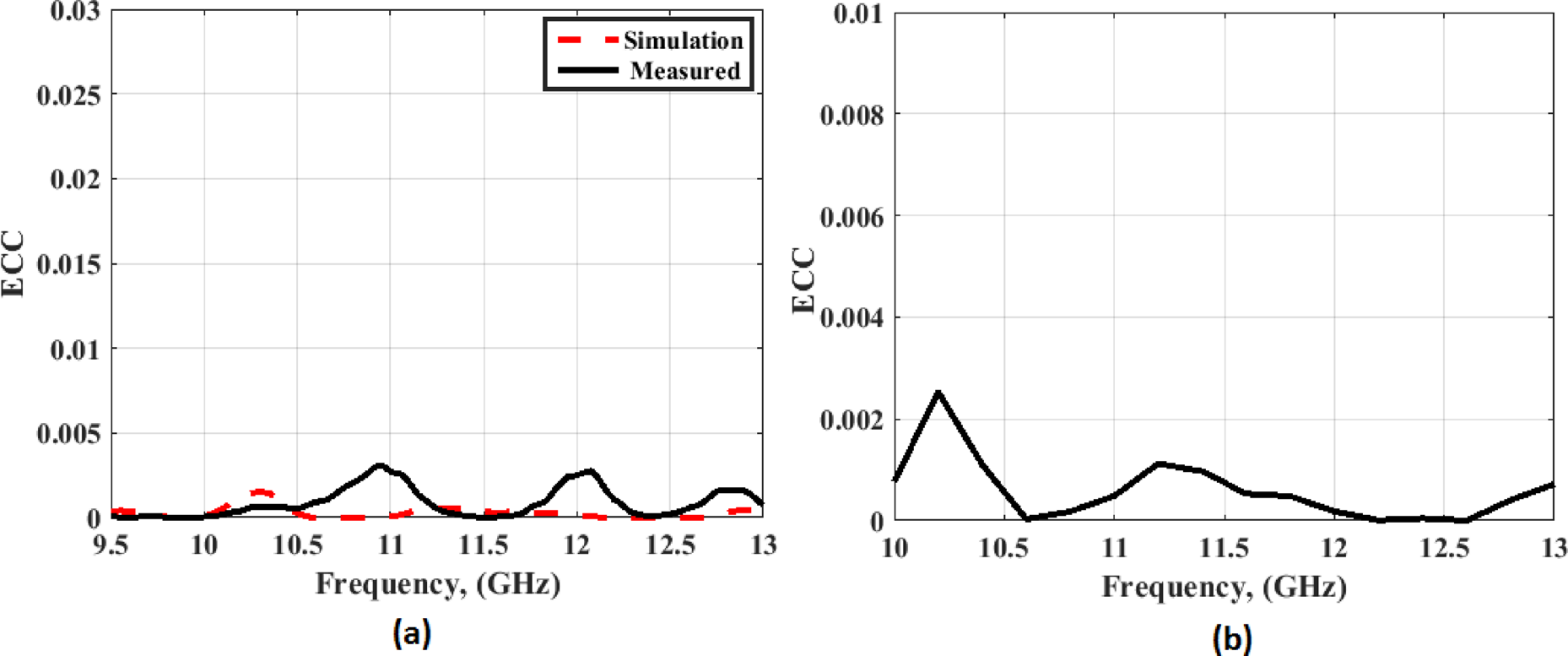
The MIMO antenna ECC at port 1 (**a**) From S-parameters (**b**) From radiation patterns.

**Fig. 20:**
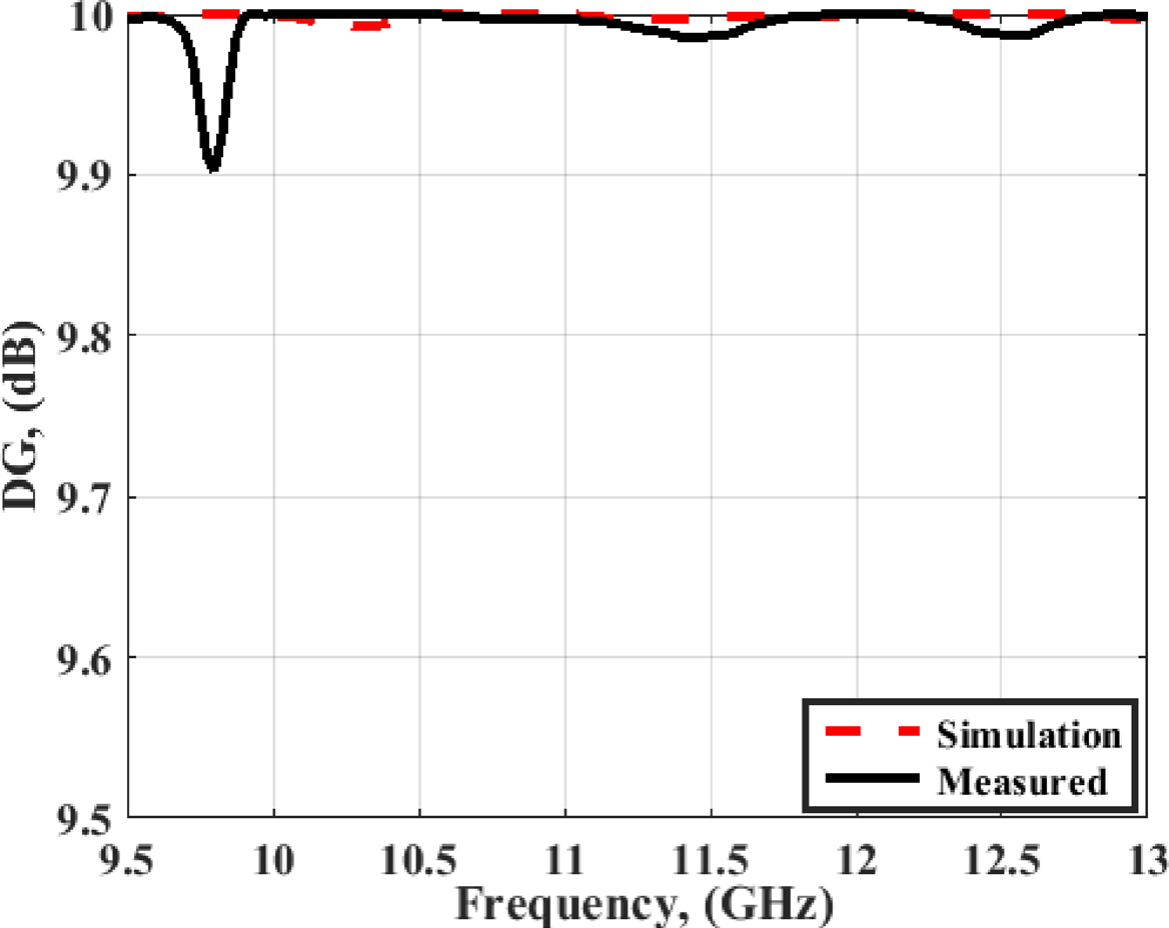
The MIMO antenna DG at port 1.

As illustrated in Fig. 18, the ECC has a value ≤ 0.002 extended within the operating frequency bands. The second parameter is the DG, which is related to the ECC from Eq. (2)^31^. The DG outcome is introduced in Fig. 20.

2 $$DG = 10 \times \sqrt {1 - {{\left| {ECC} \right|}^2}}$$

**Fig. 21:**
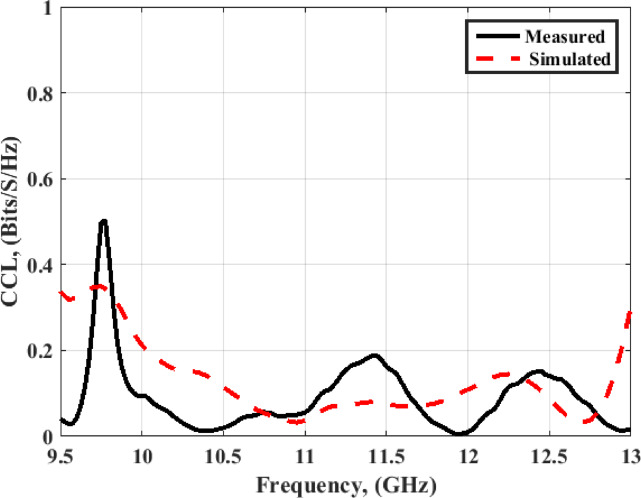
The MIMO antenna CCL at port 1.

The DG with a value ≥ 9.99 dB is achieved within the working frequency bands. As well, the CCL is also utilized to investigate the MIMO antenna performance and is calculated from Eq. (3)^32–34^, and the results are displayed in Fig. 21.

3 $$C(Loss) = - {\log _2}\det ({\psi ^R})$$

$$\begin{gathered} {\psi ^R} = \left[ {\begin{array}{*{20}{c}} {{\rho _{11}}}&{{\rho _{12}}}&{{\rho _{13}}}&{{\rho _{14}}} \\ {{\rho _{21}}}&{{\rho _{22}}}&{{\rho _{23}}}&{{\rho _{24}}} \\ {{\rho _{31}}}&{{\rho _{32}}}&{{\rho _{33}}}&{{\rho _{34}}} \\ {{\rho _{41}}}&{{\rho _{42}}}&{{\rho _{43}}}&{{\rho _{44}}} \end{array}} \right],{\rho _{ii}} = 1 - \sum\limits_{n = 1}^4 {{{\left| {{S_{in}}} \right|}^2}} \hfill \\ and \hfill \\ {\rho _{ij}} = - \left| {\sum\limits_{n = 1}^4 {S_{in}^*{S_{nj}}} } \right|,fori,j = 1,2,3or4 \hfill \\ \end{gathered}$$

The CCL with a value of ≤ 0.2 bit/s/Hz is accomplished within the achieved frequency band. From the previous discussion, it can be confirmed that the designed antenna can operate for X-band communications, and for justification purposes, the suggested four-port MIMO antenna is compared with reported antennas^19–28^ in terms of crucial parameters such as size, operating bandwidth, 3-dB AR, isolation, gain, and circular polarization availability as demonstrated in Table 1. It is observed that the suggested antenna has a compact size, high gain, and high isolation, which suggests the antenna can be used in X-band communication.

**Table 1:** Comparison of the suggested MIMO CP antenna and recently reported antennas.

Ref.	Antenna Size (λ_o_)	f_*r*_ (GHz)/ BW (%)	AR (GHz)/ BW (%)	Isolation (dB)	Gain (dBi)	Ports	CP	Connected Grounds
^19^ 2023	1.36 × 0.94 × 0.05	5.1–5.7/(9.3%)	5.1–5.7/(11.1%)	≥ 30	8	2	Yes	Yes
^20^ 2022	2.47 × 2.47 × 0.07	4.66–6.31/(30%)	4.7–6.3/(29.1%)	≥ 14	16.3	4	Yes	Yes
^21^ 2021	0.6 × 0.6 × 0.07	0.902–0.928/(10.9%)	0.83–0.96/(14.2%)	≥ 20	4.1	4	Yes	Yes
		2.4–2.485/(9.4%)	2.37–2.55/(7.4%)	≥ 25	7.4			
^22^ 2022	1.41 × 0.97 × 0.05	5.0–5.6 (11.3%)	5.0–5.6 (11.3%)	≥ 32	6-8.5	2	Yes	Yes
^23^ 2023	1.2 × 0.6 × 0.008	3–4.2 (31.5%)	2.6–3.9 (40%)	≥ 25	4	2	Yes	Yes
^24^ 2024	1.26 × 1.52 × 0.291	7.6–11.6 (43%)	9.2–9.8 (6.2%)	≥ 20	5.5–15	2	Yes	Yes
^26^ 2025	1.06 × 0.65 × 0.03	2.43–2.485 (1.6%)	2.43–2.485 (1.6%)	≥ 10	8	2	Yes	Yes
^27^ 2025	2.04 × 2.04 × 0.06	5.5–8.0 (37.1%)	6.0 to 6.55 (12.2%)	≥ 21	5.1	2	Yes	Yes
^28^ 2025	1.96 × 1.96 × 0.022	2–11.2 (139.39%)	2.25–6.4 (95.95%)	≥ 21.7	7.87	4	Yes	Yes
This work	0.98 × 0.98 × 0.385	9.8–13/(28%)	10.7–12.27/(13.67%)	≥ 20	7–8	4	Yes	Yes

## Conclusion

In the presented work, an aperture-coupled quad-port MIMO antenna is investigated to obtain the circular polarization capability for X-band wireless applications. The suggested antenna succeeded in achieving the required impedance and radiation characteristics when the simulation results were experimentally validated. An IBW of 9.8–13 GHz and a 3-dB ARBW of 10.7–12.27 GHz are reported in the manuscript, with mutual coupling between ports less than − 20 dB, and the gain varies from 7 to 8 dBi for the obtained frequency band. A diversity performance is evident when the simulated and measured MIMO parameters are incorporated, such as ECC ≤ 0.002, DG (≥ 9.99 dB), and CCL (≤ 0.2 bit/s/Hz). Novelty justification is evident when the suggested four-port MIMO antenna is compared in terms of vital parameters with the state-of-the-art, confirming its adoptability for futuristic wireless communication applications in X-band.

## Data Availability

All data generated or analyzed during this study are included in this article.

## References

[1] Ding, Z. et al. Low-profile miniaturized wideband circularly polarized monopole and MIMO antennas using characteristic mode analysis for wireless communication. *Radio Sci.* **59** (9), 1–16 (2024).

[2] Li, Z., Liu, Y., Zhao, M., Zong, W. & He, S. A wideband circularly polarized metasurface antenna with high gain using characteristic mode analysis. *Electronics* **14** (14), 2818 (2025).

[3] Nawar, R. I., Hassan, A. Y. & Abdelhady, A. M. High gain wideband circularly polarized antenna with modified ground plane. *Indonesian J. Electr. Eng. Comput. Sci.* **32** (1), 284–291 (2023).

[4] Tashvigh, V. & Kartal, M. A dual-sense CP MIMO antenna using decoupling structure with improved isolation. *AEU-International J. Electron. Commun.* **175**, 155065 (2024).

[5] Jiang, T., Jiao, T. & Li, Y. A low mutual coupling MIMO antenna using periodic multi-layered electromagnetic band gap structures. *Appl. Comput. Electromagnet. Soc. J. (ACES)* **33** (3), 305–311 (2018).

[6] Gao, S., Jiang, H., Xie, Y. F. Y. & Jiang, H. and Peiyu Wu. Analysis of scattering characteristics based on Crank-Nicolson Direct-Splitting algorithm for remote sensing problems. *IEEE Open. J. Antennas Propag.* (2024).

[7] Ibrahim, A. A., Zahra, H., Abbas, S. M., Ahmed, M. I. & Varshney, G. Subhas mukhopadhyay, and Abdelhady mahmoud. Compact four-port circularly polarized MIMO X-band DRA. *Sensors* **22** (12), 4461 (2022). 35746243 10.3390/s22124461 PMC9230936

[8] Zhang, Y. et al. Propagation characteristics of circularly and linearly polarized electromagnetic waves in urban macrocell scenario. *IEEE Trans. Veh. Technol.* **64** (1), 209–222 (2014).

[9] Malar, K. A. & Ganesh, R. S. Novel aperture coupled fractal antenna for internet of wearable things (IoWT). *Measurement: Sensors* **24**, 100533 (2022).

[10] Anandkumar, D. & Sangeetha, R. G. Design and analysis of aperture coupled micro strip patch antenna for radar applications. *Int. J. Intell. Networks*. **1**, 141–147 (2020).

[11] Mahmoud, A., Ahmed, M. I., Varshney, G. & Ibrahim, A. A. An array of staircase-shaped circularly polarized DRA. *Int. J. RF Microw. Comput. Aided Eng.* **31**, e22638. 10.1002/mmce.22638 (2021).

[12] Abdelhady, M. A., Noha, A. & Shaymaa, G. Circularly polarized chamfer-shaped DRA array, *34th National Radio Science Conference (NRSC)*, Alexandria, Egypt, 2017, pp. 38–42, Alexandria, Egypt, 2017, pp. 38–42, (2017). 10.1109/NRSC.2017.7893474

[13] Mahmoud, A. & Attia, H. Wide-band circularly polarized dielectric resonator antenna array, *IEEE International Symposium on Antennas and Propagation & USNC/URSI National Radio Science Meeting*, San Diego, CA, USA, 2017, pp. 1521–1522, San Diego, CA, USA, 2017, pp. 1521–1522 (2017).

[14] Subhanrao Bhadade R. & Padmakar Mahajan, S. Circularly polarized 4 × 4 MIMO antenna for WLAN applications. *Electromagnetics* **39 (**5), 325–342 (2019).

[15] Raj, T., Mishra, R., Kumar, P. & Kapoor, A. Advances in MIMO antenna design for 5G: A comprehensive review. *Sensors* **23** (14), 6329 (2023). 10.3390/s23146329 PMC10384345 37514623

[16] Naseri, P., Matos, S. A., Costa, J. R., Fernandes, C. A. & Nelson, J. G. F. Dual-band dual-linear-to-circular polarization converter in transmission mode application to $ k/ka $-band satellite communications. *IEEE Trans. Antennas Propag.* **66** (12), 7128–7137 (2018).

[17] Taher, F. et al. Mohamed Fathy Abo Sree, and Sara Yehia Abdel Fatah. Design and analysis of circular polarized two-port MIMO antennas with various antenna element orientations. *Micromachines* **14** (2), 380 (2023). 10.3390/mi14020380 PMC9959551 36838080

[18] Chen, H., Nerng, J. M., Song & Jung-Dong Park A compact circularly polarized MIMO dielectric resonator antenna over electromagnetic band-gap surface for 5G applications. *IEEE Access.* **7**, 140889–140898 (2019).

[19] Guthi, S. High gain and broadband circularly polarized antenna using metasurface and CPW fed L-shaped aperture. *AEU-International J. Electron. Commun.* **146**, 154109 (2022).

[20] Kim-Thi, P. Circularly polarized MIMO patch antenna with high isolation and wideband characteristics for WLAN applications. *Heliyon* **9** (9) (2023). 10.1016/j.heliyon.2023.e19450 PMC10472242 37662823

[21] Ta, S., Van Xuat, C., Nguyen, B. T. & Nguyen-Thi Thai Bao hoang, an Ngoc nguyen, Khac Kiem nguyen, and Chien Dao-Ngoc. Wideband dual-circularly polarized antennas using aperture-coupled stacked patches and single-section hybrid coupler. *IEEE Access.* **10**, 21883–21891 (2022).

[22] Zhang, E. and Jinghui Qiu. Compact four-port dual-sense circularly polarized stack-up patch antenna for UHF/MW‐RFID MIMO system. *Int. J. Antennas Propag.* **1** (2021), 2118059 (2021).

[23] Hussain, N., Pham, T. D. & Huy-Hung, T. Circularly polarized MIMO antenna with wideband and high isolation characteristics for C-band communication systems. *Micromachines* **13 ** (11), 1894 (2022). 10.3390/mi13111894 PMC9694123 36363915

[24] Dwivedi, A., Kumar, N. K. & Narayanaswamy Krishna Kanth varma penmatsa, Suyash Kumar singh, Anand sharma, and Vivek singh. Circularly polarized printed dual Port MIMO antenna with polarization diversity optimized by machine learning approach for 5G NR n77/n78 frequency band applications. *Sci. Rep.* **13** (1), 13994 (2023). 37634021 10.1038/s41598-023-41302-2 PMC10460397

[25] Singh, A., Kumar, A. & Binod Kumar Kanaujia FSS inspired two Port CP MIMO antenna with enhanced gain for X-band applications. *Opt. Commun.* **566**, 130698 (2024).

[26] Tran-Huy, D., Nguyen-Viet-Duc, T. & Tran, H. Hussain, N. A compact MIMO antenna with high gain and dual circular polarization using a T divider for WLAN applications. *Sci. Rep.* **15**, 22106 (2025). 40594619 10.1038/s41598-025-05820-5 PMC12218960

[27] Iqbal, J., Illahi, U., Ramay, S. M. & Sulaiman, M. I. Circularly polarized dual-port MIMO DRA for future Wi-Fi 6E applications. *Int. J. Microw. Wirel. Technol.* **16**, 1236–1247 (2024).

[28] Nawar, R. I., Abdelhady, A. M. & Hassan, A. Y. High performance circularly polarized quad-elements for 5G wireless communications. *Arab. J. Sci. Eng. ***50**, 10837–10854 (2025).

[29] Kamal, M. M. et al. Self-decoupled quad‐port CPW‐fed fractal MIMO antenna with UWB characteristics. *Int. J. Antennas Propag.* **2024(1)**, 3826899 (2024).

[30] Ahmed, M., Ahmed, M. I. & Ibrahim, A. A. and Shaymaa M. Gaber. Quad-port 28/38 ghz antenna with isolation improvement for 5G wireless networks. *J. Infrared Millim. Terahertz Waves ***45**, 672–693 (2024).

[31] Sharma, P., Tiwari, R. N., Singh, P., Kumar, P. & Kanaujia, B. K. MIMO antennas: design approaches, techniques and applications. *Sensors* **22** (20), 7813 (2022). 36298163 10.3390/s22207813 PMC9608594

[32] Desai, A. et al. Interconnected CPW fed flexible 4-port MIMO antenna for UWB, X, and Ku band applications. *IEEE Access.* **10**, 57641–57654 (2022).

[33] Upadhyaya, T. et al. Quad-port MIMO antenna with high isolation characteristics for sub 6-GHz 5G NR communication. *Sci. Rep.* **13** (1), 19088 (2023). 37925589 10.1038/s41598-023-46413-4 PMC10625610

[34] Upadhyaya, T. et al. Gangil Byun, and Yogeshwar Kosta. Aperture-fed quad-port dual-band dielectric resonator MIMO antenna for sub-6 GHz 5G and WLAN Application. *Int.l J. Antennas Propag.* **1** (2022), 4136347 (2022).

